# *N*′-[4-(Dimethyl­amino)­benzyl­idene]-4-meth­oxybenzohydrazide

**DOI:** 10.1107/S1600536808031012

**Published:** 2008-09-30

**Authors:** Jiu-Fu Lu

**Affiliations:** a School of Chemistry and Environmental Science, Shaanxi University of Technology, Hanzhong 723000, People’s Republic of China

## Abstract

The title Schiff base compound, C_17_H_19_N_3_O_2_, was obtained from the condensation of 4-dimethyl­amino­benzaldehyde with 4-methoxy­benzohydrazide in an ethanol solution. The mol­ecule is twisted with respect to the N—N single bond [C—N—N—C = −159.27 (14)°] and the dihedral angle between the two aromatic rings is 67.1 (2)°. In the crystal structure, the mol­ecules are linked into chains along the *c* axis by inter­molecular N—H⋯O and C—H⋯O hydrogen bonds.

## Related literature

For related structures, see: Lu *et al.* (2008*a* ▶,*b* ▶,*c* ▶); Nie (2008 ▶); He (2008 ▶); Shi *et al.* (2007 ▶). For bond-length data, see: Allen *et al.* (1987 ▶).
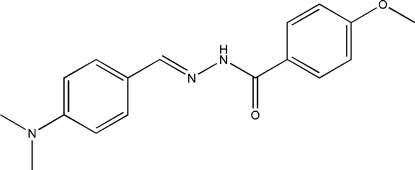


## Experimental

### 

#### Crystal data

C_17_H_19_N_3_O_2_
*M* *_r_* = 297.35
Monoclinic, 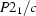

*a* = 11.922 (4) Å
*b* = 13.224 (5) Å
*c* = 9.756 (4) Å
β = 91.469 (6)°
*V* = 1537.6 (10) Å^3^
*Z* = 4
Mo *K*α radiation
μ = 0.09 mm^−1^
*T* = 298 (2) K
0.23 × 0.23 × 0.20 mm

#### Data collection

Bruker APEXII CCD area-detector diffractometer
Absorption correction: multi-scan (*SADABS*; Sheldrick, 2004 ▶) *T*_min_ = 0.981, *T*_max_ = 0.983
8744 measured reflections
3296 independent reflections
2507 reflections with *I* > 2σ(*I*)
*R*_int_ = 0.020

#### Refinement

*R*[*F* ^2^ > 2σ(*F* ^2^)] = 0.043
*wR*(*F* ^2^) = 0.121
*S* = 1.03
3296 reflections
205 parameters
1 restraint
H atoms treated by a mixture of independent and constrained refinement
Δρ_max_ = 0.14 e Å^−3^
Δρ_min_ = −0.18 e Å^−3^

### 

Data collection: *APEX2* (Bruker, 2004 ▶); cell refinement: *SAINT* (Bruker, 2004 ▶); data reduction: *SAINT*; program(s) used to solve structure: *SHELXS97* (Sheldrick, 2008 ▶); program(s) used to refine structure: *SHELXL97* (Sheldrick, 2008 ▶); molecular graphics: *SHELXTL* (Sheldrick, 2008 ▶); software used to prepare material for publication: *SHELXTL*.

## Supplementary Material

Crystal structure: contains datablocks global, I. DOI: 10.1107/S1600536808031012/ci2684sup1.cif

Structure factors: contains datablocks I. DOI: 10.1107/S1600536808031012/ci2684Isup2.hkl

Additional supplementary materials:  crystallographic information; 3D view; checkCIF report

## Figures and Tables

**Table 1:** Hydrogen-bond geometry (Å, °)

*D*—H⋯*A*	*D*—H	H⋯*A*	*D*⋯*A*	*D*—H⋯*A*
N2—H2*A*⋯O1^i^	0.90 (1)	1.99 (1)	2.873 (2)	167 (2)
C7—H7⋯O1^i^	0.93	2.54	3.297 (2)	139

Symmetry code: (i) 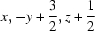.

**Fig. 1.**
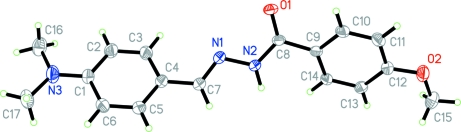
The molecular structure of the title compound, showing 30% probability displacement ellipsoids and the atom-numbering scheme.

**Fig. 2.**
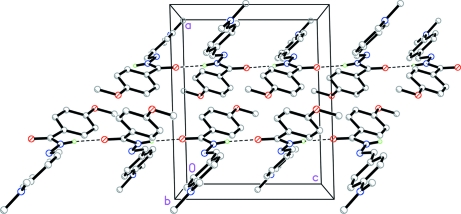
The crystal packing of the title compound, showing N—H···O hydrogen-bonded (dashed lines) chains running along the c axis. C-bound H atoms have been omitted for clarity.

## supplementary crystallographic information

### Comment

As part of our investigation of the crystal structures of Schiff bases derived from the condensation of aldehydes with benzohydrazides (Lu *et al.*, 2008*a*,*b*,*c*), we report here the crystal structure of the title new Schiff base compound.

In the title molecule (Fig. 1), the bond lengths have normal values (Allen *et al.*, 1987), and are comparable to those observed in similar compounds (Nie, 2008; He, 2008; Shi *et al.*, 2007). The dihedral angle between the two aromatic rings is 67.1 (2)°, indicating that the molecule is twisted. The methoxy group is coplanar with the attached ring [C15—O2—C12—C13 = 0.8 (2)°]. The dimethylamino group is almost coplanar with the attached ring [C16—N3—C1—C2 = 3.8 (2)° and C17—N3—C1—C6 = 8.7 (3)°]. The C7–N1—N2—C8 torsion angle [-159.27 (14)°] indicates that the molecule is twisted about the N1—N2 bond.

In the crystal structure, the molecules are linked into chains (Fig. 2) running along the *c* axis by intermolecular N—H···O and C—H···O hydrogen bonds (Table 1).

### Experimental

The title compound was prepared by the Schiff base condensation of 4-dimethylaminobenzaldehyde (0.1 mol) and 4-methoxybenzohydrazide (0.1 mol) in 95% ethanol (50 ml). The excess ethanol was removed by distillation. The colourless solid obtained was filtered and washed with ethanol. Single crystals suitable for X-ray diffraction were obtained by slow evaporation of a 95% ethanol solution at room temperature.

### Refinement

The imino H atom was located in a difference map and refined with a N—H distance restraint of 0.90 (1) Å and a fixed U_iso_ of 0.08 Å^2^. The other H atoms were positioned geometrically (C—H = 0.93–0.96 Å) and refined using a riding model, with U_iso_(H) = 1.2U_eq_(C) and 1.5U_eq_(C_methyl_). A rotating group model was used for methyl groups.

### Crystal data

**Table:**

C_17_H_19_N_3_O_2_	*F*(000) = 632
*M**_r_* = 297.35	*D*_x_ = 1.284 Mg m^−^^3^
Monoclinic, *P*2_1_/*c*	Mo *K*α radiation, λ = 0.71073 Å
Hall symbol: -P 2ybc	Cell parameters from 3161 reflections
*a* = 11.922 (4) Å	θ = 2.5–27.2°
*b* = 13.224 (5) Å	µ = 0.09 mm^−^^1^
*c* = 9.756 (4) Å	*T* = 298 K
β = 91.469 (6)°	Block, colourless
*V* = 1537.6 (10) Å^3^	0.23 × 0.23 × 0.20 mm
*Z* = 4	

### Data collection

**Table:**

Bruker APEXII CCD area-detector diffractometer	3296 independent reflections
Radiation source: fine-focus sealed tube	2507 reflections with *I* > 2σ(*I*)
graphite	*R*_int_ = 0.020
ω scans	θ_max_ = 27.0°, θ_min_ = 2.3°
Absorption correction: multi-scan (SADABS; Sheldrick, 2004)	*h* = −14→14
*T*_min_ = 0.981, *T*_max_ = 0.983	*k* = −16→14
8744 measured reflections	*l* = −12→12

### Refinement

**Table:**

Refinement on *F*^2^	Primary atom site location: structure-invariant direct methods
Least-squares matrix: full	Secondary atom site location: difference Fourier map
*R*[*F*^2^ > 2σ(*F*^2^)] = 0.043	Hydrogen site location: inferred from neighbouring sites
*wR*(*F*^2^) = 0.121	H atoms treated by a mixture of independent and constrained refinement
*S* = 1.03	*w* = 1/[σ^2^(*F*_o_^2^) + (0.0597*P*)^2^ + 0.2298*P*] where *P* = (*F*_o_^2^ + 2*F*_c_^2^)/3
3296 reflections	(Δ/σ)_max_ = 0.001
205 parameters	Δρ_max_ = 0.15 e Å^−^^3^
1 restraint	Δρ_min_ = −0.18 e Å^−^^3^

### Special details

**Table:**

Geometry. All esds (except the esd in the dihedral angle between two l.s. planes) are estimated using the full covariance matrix. The cell esds are taken into account individually in the estimation of esds in distances, angles and torsion angles; correlations between esds in cell parameters are only used when they are defined by crystal symmetry. An approximate (isotropic) treatment of cell esds is used for estimating esds involving l.s. planes.
Refinement. Refinement of F^2^ against ALL reflections. The weighted R-factor wR and goodness of fit S are based on F^2^, conventional R-factors R are based on F, with F set to zero for negative F^2^. The threshold expression of F^2^ > 2sigma(F^2^) is used only for calculating R-factors(gt) etc. and is not relevant to the choice of reflections for refinement. R-factors based on F^2^ are statistically about twice as large as those based on F, and R- factors based on ALL data will be even larger.

### Fractional atomic coordinates and isotropic or equivalent isotropic displacement parameters (Å^2^)

**Table:**

	*x*	*y*	*z*	*U*_iso_*/*U*_eq_	
N1	0.23023 (11)	0.86331 (9)	0.18617 (12)	0.0451 (3)	
N2	0.27424 (11)	0.77478 (9)	0.24111 (11)	0.0441 (3)	
N3	0.06781 (13)	1.32361 (10)	0.12512 (17)	0.0665 (4)	
O1	0.29961 (10)	0.70488 (7)	0.03342 (9)	0.0520 (3)	
O2	0.46299 (11)	0.33676 (8)	0.39093 (12)	0.0612 (3)	
C1	0.10930 (12)	1.23056 (11)	0.15842 (16)	0.0473 (4)	
C2	0.07206 (14)	1.14376 (12)	0.09023 (18)	0.0552 (4)	
H2	0.0181	1.1495	0.0201	0.066*	
C3	0.11325 (14)	1.05048 (11)	0.12429 (17)	0.0517 (4)	
H3	0.0874	0.9943	0.0757	0.062*	
C4	0.19240 (12)	1.03716 (10)	0.22919 (14)	0.0428 (3)	
C5	0.22870 (14)	1.12314 (11)	0.29766 (16)	0.0523 (4)	
H5	0.2809	1.1166	0.3696	0.063*	
C6	0.19031 (14)	1.21761 (12)	0.26300 (17)	0.0545 (4)	
H6	0.2185	1.2738	0.3096	0.065*	
C7	0.23746 (12)	0.93903 (11)	0.26614 (15)	0.0440 (3)	
H7	0.2730	0.9311	0.3514	0.053*	
C8	0.30424 (12)	0.69893 (10)	0.15876 (13)	0.0389 (3)	
C9	0.34473 (11)	0.60594 (10)	0.22874 (13)	0.0371 (3)	
C10	0.41496 (13)	0.54231 (11)	0.15756 (14)	0.0468 (4)	
H10	0.4359	0.5597	0.0695	0.056*	
C11	0.45393 (14)	0.45430 (11)	0.21489 (16)	0.0506 (4)	
H11	0.5018	0.4129	0.1661	0.061*	
C12	0.42254 (13)	0.42666 (10)	0.34493 (15)	0.0442 (3)	
C13	0.35380 (13)	0.48939 (11)	0.41792 (14)	0.0452 (3)	
H13	0.3334	0.4719	0.5062	0.054*	
C14	0.31545 (12)	0.57821 (10)	0.35951 (14)	0.0421 (3)	
H14	0.2689	0.6203	0.4091	0.051*	
C15	0.43137 (17)	0.30433 (13)	0.52240 (19)	0.0676 (5)	
H15A	0.4527	0.3546	0.5891	0.101*	
H15B	0.4683	0.2417	0.5444	0.101*	
H15C	0.3516	0.2947	0.5230	0.101*	
C16	−0.02077 (16)	1.33522 (15)	0.0240 (2)	0.0727 (6)	
H16A	0.0088	1.3263	−0.0658	0.109*	
H16B	−0.0526	1.4016	0.0311	0.109*	
H16C	−0.0778	1.2855	0.0391	0.109*	
C17	0.1166 (2)	1.41355 (13)	0.1829 (2)	0.0810 (6)	
H17A	0.1116	1.4115	0.2809	0.121*	
H17B	0.0768	1.4716	0.1481	0.121*	
H17C	0.1939	1.4178	0.1584	0.121*	
H2A	0.2875 (17)	0.7721 (14)	0.3316 (10)	0.080*	

### Atomic displacement parameters (Å^2^)

**Table:**

	*U*^11^	*U*^22^	*U*^33^	*U*^12^	*U*^13^	*U*^23^
N1	0.0607 (8)	0.0397 (6)	0.0349 (6)	0.0055 (5)	−0.0015 (5)	0.0055 (5)
N2	0.0648 (8)	0.0385 (6)	0.0289 (6)	0.0075 (6)	−0.0026 (5)	0.0025 (5)
N3	0.0689 (10)	0.0429 (7)	0.0867 (11)	0.0069 (7)	−0.0189 (8)	0.0055 (7)
O1	0.0804 (8)	0.0482 (6)	0.0272 (5)	−0.0012 (5)	−0.0051 (5)	0.0000 (4)
O2	0.0753 (8)	0.0472 (6)	0.0613 (7)	0.0160 (5)	0.0055 (6)	0.0097 (5)
C1	0.0475 (8)	0.0404 (8)	0.0539 (9)	0.0031 (6)	0.0005 (7)	0.0035 (7)
C2	0.0551 (9)	0.0500 (9)	0.0595 (10)	−0.0008 (7)	−0.0172 (8)	0.0033 (7)
C3	0.0562 (9)	0.0421 (8)	0.0562 (10)	−0.0031 (7)	−0.0117 (7)	−0.0015 (7)
C4	0.0487 (8)	0.0404 (7)	0.0393 (7)	0.0024 (6)	0.0020 (6)	0.0027 (6)
C5	0.0606 (10)	0.0483 (9)	0.0473 (9)	0.0059 (7)	−0.0118 (7)	−0.0031 (7)
C6	0.0633 (10)	0.0424 (8)	0.0570 (10)	0.0023 (7)	−0.0099 (8)	−0.0065 (7)
C7	0.0525 (8)	0.0438 (8)	0.0357 (7)	0.0028 (6)	−0.0004 (6)	0.0037 (6)
C8	0.0482 (8)	0.0403 (7)	0.0279 (7)	−0.0055 (6)	−0.0018 (5)	−0.0009 (5)
C9	0.0462 (7)	0.0360 (7)	0.0291 (6)	−0.0029 (6)	−0.0012 (5)	−0.0028 (5)
C10	0.0597 (9)	0.0479 (8)	0.0332 (7)	0.0016 (7)	0.0091 (6)	−0.0009 (6)
C11	0.0598 (9)	0.0469 (8)	0.0457 (8)	0.0093 (7)	0.0110 (7)	−0.0053 (7)
C12	0.0491 (8)	0.0379 (7)	0.0453 (8)	0.0026 (6)	−0.0012 (6)	0.0003 (6)
C13	0.0572 (9)	0.0453 (8)	0.0334 (7)	0.0016 (7)	0.0048 (6)	0.0045 (6)
C14	0.0537 (8)	0.0393 (7)	0.0337 (7)	0.0057 (6)	0.0061 (6)	−0.0019 (6)
C15	0.0793 (12)	0.0550 (10)	0.0684 (12)	0.0059 (9)	−0.0023 (9)	0.0231 (9)
C16	0.0609 (11)	0.0672 (11)	0.0896 (15)	0.0183 (9)	−0.0083 (10)	0.0133 (10)
C17	0.1003 (16)	0.0422 (9)	0.0996 (16)	0.0101 (10)	−0.0138 (13)	−0.0015 (10)

### Geometric parameters (Å, °)

**Table:**

N1—C7	1.2710 (18)	C7—H7	0.93
N1—N2	1.3851 (16)	C8—C9	1.4814 (19)
N2—C8	1.3395 (17)	C9—C14	1.3810 (19)
N2—H2A	0.893 (9)	C9—C10	1.386 (2)
N3—C1	1.3624 (19)	C10—C11	1.367 (2)
N3—C17	1.433 (2)	C10—H10	0.93
N3—C16	1.435 (2)	C11—C12	1.381 (2)
O1—C8	1.2252 (16)	C11—H11	0.93
O2—C12	1.3550 (17)	C12—C13	1.377 (2)
O2—C15	1.413 (2)	C13—C14	1.3784 (19)
C1—C2	1.394 (2)	C13—H13	0.93
C1—C6	1.397 (2)	C14—H14	0.93
C2—C3	1.366 (2)	C15—H15A	0.96
C2—H2	0.93	C15—H15B	0.96
C3—C4	1.385 (2)	C15—H15C	0.96
C3—H3	0.93	C16—H16A	0.96
C4—C5	1.383 (2)	C16—H16B	0.96
C4—C7	1.447 (2)	C16—H16C	0.96
C5—C6	1.370 (2)	C17—H17A	0.96
C5—H5	0.93	C17—H17B	0.96
C6—H6	0.93	C17—H17C	0.96
			
C7—N1—N2	114.15 (12)	C10—C9—C8	117.84 (12)
C8—N2—N1	120.33 (11)	C11—C10—C9	121.00 (13)
C8—N2—H2A	121.3 (13)	C11—C10—H10	119.5
N1—N2—H2A	118.2 (13)	C9—C10—H10	119.5
C1—N3—C17	120.98 (15)	C10—C11—C12	120.25 (14)
C1—N3—C16	121.12 (15)	C10—C11—H11	119.9
C17—N3—C16	117.75 (15)	C12—C11—H11	119.9
C12—O2—C15	117.78 (13)	O2—C12—C13	124.65 (14)
N3—C1—C2	121.39 (14)	O2—C12—C11	115.72 (13)
N3—C1—C6	121.65 (14)	C13—C12—C11	119.63 (13)
C2—C1—C6	116.96 (13)	C12—C13—C14	119.67 (13)
C3—C2—C1	121.31 (14)	C12—C13—H13	120.2
C3—C2—H2	119.3	C14—C13—H13	120.2
C1—C2—H2	119.3	C13—C14—C9	121.28 (13)
C2—C3—C4	121.90 (14)	C13—C14—H14	119.4
C2—C3—H3	119.0	C9—C14—H14	119.4
C4—C3—H3	119.0	O2—C15—H15A	109.5
C5—C4—C3	116.82 (13)	O2—C15—H15B	109.5
C5—C4—C7	120.51 (13)	H15A—C15—H15B	109.5
C3—C4—C7	122.66 (13)	O2—C15—H15C	109.5
C6—C5—C4	122.15 (15)	H15A—C15—H15C	109.5
C6—C5—H5	118.9	H15B—C15—H15C	109.5
C4—C5—H5	118.9	N3—C16—H16A	109.5
C5—C6—C1	120.82 (15)	N3—C16—H16B	109.5
C5—C6—H6	119.6	H16A—C16—H16B	109.5
C1—C6—H6	119.6	N3—C16—H16C	109.5
N1—C7—C4	122.33 (13)	H16A—C16—H16C	109.5
N1—C7—H7	118.8	H16B—C16—H16C	109.5
C4—C7—H7	118.8	N3—C17—H17A	109.5
O1—C8—N2	123.01 (13)	N3—C17—H17B	109.5
O1—C8—C9	121.27 (12)	H17A—C17—H17B	109.5
N2—C8—C9	115.72 (11)	N3—C17—H17C	109.5
C14—C9—C10	118.16 (13)	H17A—C17—H17C	109.5
C14—C9—C8	124.00 (12)	H17B—C17—H17C	109.5

### Hydrogen-bond geometry (Å, °)

**Table:**

*D*—H···*A*	*D*—H	H···*A*	*D*···*A*	*D*—H···*A*
N2—H2A···O1^i^	0.90 (1)	1.99 (1)	2.873 (2)	167 (2)
C7—H7···O1^i^	0.93	2.54	3.297 (2)	139

Symmetry codes: (i) *x*, −*y*+3/2, *z*+1/2.
